# Biosynthesis of Silver Nanoparticles From Marine Actinobacterium Micromonospora sp. and Their Bioactive Potential

**DOI:** 10.7759/cureus.53870

**Published:** 2024-02-08

**Authors:** Keshav Rajesh, Sivaperumal Pitchiah, Kamala Kannan, Vasugi Suresh

**Affiliations:** 1 Physiology, Saveetha Dental College and Hospitals, Saveetha Institute of Medical and Technical Sciences (SIMATS) Saveetha University, Chennai, IND; 2 Prosthodontics, Saveetha Dental College and Hospitals, Saveetha Institute of Medical and Technical Sciences (SIMATS) Saveetha University, Chennai, IND

**Keywords:** taxonomy, good health and wellbeing, micromonospora sp., agnps (silver nanoparticles), marine actinobacteria

## Abstract

Background

The biosynthesis of nanoparticles represents a rapid, environmentally friendly, cost-effective, and straightforward technology. This approach allows for the production of nanoparticles with a wide range of chemical compositions, sizes, shapes, high uniformity, and scalability. One of the principal advantages of biogenic nanoparticles is their water solubility and compatibility with biological systems. Biologically synthesized nanoparticles have demonstrated superior efficiency compared to conventionally synthesized particles. Among biosynthesis, microbial-mediated biosynthesis is a promising one that has a selectively reducing ability on specific metal ions through electron transfer.

Objectives

Evaluation of antimicrobial and antioxidant activity of silver nanoparticle synthesized by actinobacteria *Micromonospora* *sp.* which is isolated from marine environment.

Materials and methods

In this study, actinobacteria were isolated from the marine sediment using the spread plate method. The isolates were identified based on morphological observation, cell wall amino acids, sugar analysis, and micromorphological analysis. The silver nanoparticle synthesis from microbes and their inhibition against clinical pathogens have been evaluated by the disc diffusion method. Antioxidant efficiency was evaluated in terms of total antioxidant activity through ammonium molybdenum assay.

Results

A total of five isolates were isolated from the sediment sample. The cell-free extract of MBIT-MSA4 can synthesize silver nanoparticles that have potential antimicrobial activity against the clinical pathogens *Streptococcus mutans* at a zone of inhibition 6 mm, 10 mm inhibition zone of *Klebsiella pneumonia, *and 8 mm zone of inhibition of *Staphylococcus aureus*. Also, it has significant antioxidant activity up to 73% of free radical inhibition.

Conclusion

Marine microbial-mediated biosynthesized silver nanoparticles have potential antimicrobial activity against S. *mutans* and methicillin-resistant *Staphylococcus aureus* (MRSA) and inhibit the oxidation process through antioxidant activity. This enhanced efficient biosynthesised nanoparticle has significantly reduced the concentration of free radicals caused by pathogens.

## Introduction

Nanoparticles (AgNP), which are materials with dimensions less than 100 nm, are leading the way in the advancement of nanotechnology in the field of biomedical research [1]. These tiny particles have a notable ratio between the surface area to volume of the particles, giving them distinct features and improving their abilities in areas such as mechanics, catalysis, optics, and magnetism. This broadens their potential applications in biomedicine [2]. Among various metals, silver has been extensively used in applications such as pathogen control, water purification, and food preservation [3]. Recent developments in nanotechnology have made silver nanoparticles (AgNPs) widely used for their antimicrobial, anticancer, and anti-inflammatory properties due to their unique optical, magnetic, catalytic, and electronic features [4]. Scientific research has revealed multiple mechanisms through which AgNPs can disturb cellular metabolic functions. One of these mechanisms is the degradation of DNA through the intrusion of AgNP particles into cells, causing oxidation [2]. Therefore, the potential of AgNP for therapeutic and biotechnological applications has received significant attention in recent years [1]. AgNPs also tend to aggregate within the nucleus, mitochondria, and lysosomes, causing significant oxidative damage to pathogens [2]. Another mechanism contributing to the cytotoxicity of AgNPs to pathogens is the generation of reactive oxygen species (ROS) as a result of disrupting the mitochondrial electron transport chain, leading to DNA damage [5].

The cytotoxicity of AgNPs has gained prominence in recent research due to their ultra-small size, which facilitates easy entry into cells and transport through the bloodstream [6]. Consequently, smaller AgNPs (3-7 nm) exhibit higher cytotoxicity in mouse cells compared to larger AgNPs (10-40 nm). Additionally, AgNPs can inhibit free radicals like superoxide and hydroxyl and neutralize radicals through electron transfer reactions [7]. They can convert hydrogen peroxide into water and oxygen through catalytic activity. They can donate electrons to neutralize free radicals and interact with reactive oxygen species in the form of silver ions (Ag^+^) [8]. Over the years, various physical techniques, including thermal decomposition, microwaves, and chemical reduction have been used to synthesize AgNPs. Despite their effectiveness, these traditional methods are known for consuming large amounts of energy, using dangerous chemicals, requiring significant amounts of time, and posing potential risks to the environment and living organisms. As a result, the potential applications of AgNPs for therapeutic purposes are considerably restricted [9]. In contrast, biological methods provide simpler, quicker, and more cost-effective approaches for both synthesizing nanostructured materials and reducing the generation of environmentally and biologically harmful substances [2]. Hence, there is a shift in focus from conventional (physical and chemical) methods to "green" chemistry, specifically biosynthesis. Microorganisms play a crucial role in providing numerous nucleation centers and creating conditions beneficial for the higher yield of thoroughly dispersed particles [10]. They also immobilize particles and create a viscous medium that contains extracellular enzymes, ultimately preventing or reducing particle aggregation. This process has significant implications in various environments, as microorganisms can alter the physical properties of their surroundings [11]. Several studies have explored the use of bacteria, such as *Bacillus subtilis*, and fungi, such as *Fusarium oxysporum*, in AgNP biosynthesis [12]. Actinobacteria, resourceful gram-positive bacteria with a high guanine and cytosine (GC) content, are widespread and known for producing bioactive substances with significant biomedical and industrial applications. The bioactive compounds produced by actinobacteria possess various therapeutic properties, including antimicrobial, anticancer, and anthelmintic properties [13]. Furthermore, bioactive metabolites from actinobacteria enhance AgNP synthesis through reduction processes. Additionally, multidrug resistance (MDR) remains a persistent global challenge, necessitating the development of innovative nanomaterials for constructing new antimicrobial biomaterials. This biosynthesized bioactive nanoparticle has a higher possibility of opening up new therapeutic avenues in medical approaches [14].

Studies on the efficacy of Bio-AgNPs produced by marine actinobacteria as antimicrobial candidates against MDR pathogens and their antioxidant potentials are essential for society [15]. Therefore, this study aims to evaluate the antibacterial and antioxidant activities of silver nanoparticles that have been biosynthesized through microbial processes. The findings of this study pave the way for the production of Bio-AgNPs with potent antibacterial and antioxidant properties from marine actinobacteria.

## Materials and methods

Sample collection and isolation

Sediment samples were collected from the Chennai coast, of India at a depth (30cm) by a sterile scope and stored in sterile plastic ziplock bags. The sample bag was kept at 4^°^C until reaching the laboratory. Marine actinobacteria was isolated on specific medium actinobacterial isolation agar by spread plate method. The spread plates were stored at 37^°^C for seven to 12 days.

Identification

Five distinct colonies (MBIT-MSA1 to 5) from the spread plates were taken for identification through morphological, biochemical, and micromorphological analysis. Morphological observations such as the color of aerial mycelium and substrate mycelium on Internatonation Streptomyces Project (ISP) 2 and ISP7 medium were noted [16]. The biochemical analysis involved analyzing the cell wall amino-acid and sugar pattern using Bergey’s manual of determinative bacteriology [17]. In micromorphological analysis, the length and shape of spores were observed using light microscopy [18]. 

Silver nanoparticle synthesis

All five strains (MBIT-MSA 1 to 5) were cultivated in starch casein agar medium with various concentrations of silver nitrate, to evaluate their tolerance and resistance levels. The resistant isolates were then used for further nanoparticle synthesis. Silver nanoparticles AgNP’s were biosynthesized using the secondary metabolites of marine actinobacteria (MBIT-MSA4) as a reducing agent. Well-grown actinobacterial culture (100 ml) was taken for the extraction of secondary metabolites by centrifugation at 10,000 rpm for 30 minutes. After centrifugation, the aqueous extract was filtered (0.2 mm) through a poly-tetra-fluoroethylene (PTFE) membrane filter (SLFG02550), and the nanoparticles were resuspended in deionized water and centrifuged at 12,000 rpm for 15 min and it repeated twice to eliminate organic and other impurities. The biosynthesized silver nanoparticles were confirmed through the spectrum of UV-visible spectroscopy within the wavelength range of 300-700 nm. 

Antibacterial activity

The antibacterial activity of silver nanoparticles (AgNPs) was determined using clinical pathogens *Streptococcus mutans*, *Klebsiella pneumonia,* and *Staphylococcus aureus*. The pathogens were swabbed on a specific medium, and then a sterile disc with the sample was placed and incubated for 24 h at 37^°^C. After incubation, the zone of inhibition was noted [19].

Antioxidant activity 

The antioxidant activity of the silver nanoparticles was determined using the phospho-molybdenum method [20]. A reaction mixture was prepared with sulfuric acid (0.6 M), sodium phosphate (28 mM), and ammonium molybdate (4 mM) in 4.5 ml along with 500 ml of sample at various concentrations. After addition, the samples were incubated in a water bath at 95^°^C for one and a half hours. After incubation, the samples were cooled to room temperature, and the absorbance was measured at 695 nm using a UV spectrophotometer (Shimadzu). 

Statistical analysis

All results were statistically analyzed, and the graphs were created with the mean ± standard deviation of four replicates. The results were analyzed using SPSS (SPSS Inc. Released 2007. SPSS for Windows, Version 16.0. Chicago, SPSS Inc).

## Results

**Figure 1:**
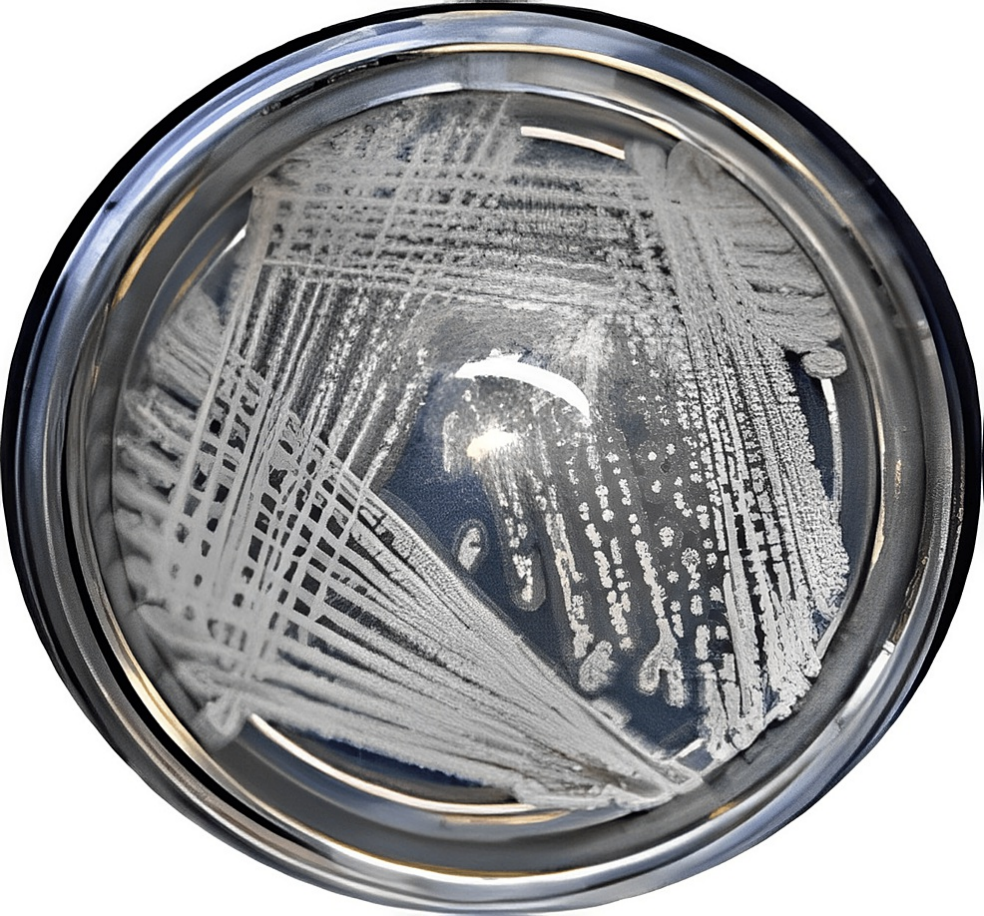
Morphology of the isolated MBIT-MSA4 Micromonospora sp.

Overall, five distinct colony morphological obtained were isolated from the sediment sample. The resistance of all five isolates to silver nitrate was analyzed and it was found that only MBIT-MSA4 exhibited resistance. These isolates were selected for further silver nanoparticle synthesis. The other four isolates did not show any resistance to silver nitrate, so the study focused on MBIT-MSA4. The potential isolate MBIT-MSA4 displayed whitish peach-colored aerial and substrate mycelium on ISP2 medium and white-colored mycelium on ISP7 medium. No soluble or melanoid pigments were produced on either ISP2 or ISP7 medium (Figure 1). 

Biochemical analysis of the cell wall reveals the presence of the amino-acid meso-diaminopimelic acid (meso-DAP) along with glycine and other amino-acids such as L-ornithine, lysine, and LL-di-amino-pimelic acid (LL-DAP) does not appear in the results (Table 1). These findings suggest that the cell wall belongs to type II. 

**Table 1:** Biochemical analysis of cell wall amino-acid analysis of MBIT-MSA4 LL-DAP = LL-DiAminopimelic acid; meso-DAP=meso-diaminopimelic acid; + = Present; - = Absent.

CELL WALL AMINO ACID ANALYSIS	
LL-DAP	-
meso-DAP	+
GLYCINE	+
LYSINE	-
L-ORNITHINE	-
INFERENCE	TYPE II

The isolate MBIT-MSA4 contains arabinose and xylose in its cell wall, while madurose, galactose, and rhamnose were present in lower levels that could not be quantified (Table 2). Therefore the results indicate a significant sugar content in the cell wall suggesting pattern D.

**Table 2:** Cell wall sugar pattern analysis of MBIT-MSA4 + = Present; - = Absent.

SUGAR PATTERN ANALYSIS	
ARABINOSE	+
MADUROSE	-
GALACTOSE	-
XYLOSE	+
RHAMNOSE	-
INFERENCE	D

The combined taxonomical results of amino acid type II with sugar pattern D and mono spores have inferred that the isolate has the possibility of belonging to the genus *Micromonospora* *sp*. The combination results of morphological observation, biochemical observation, and spore chain morphology have confirmed that the isolate is a species of *Micromonospora*.

**Figure 2:**
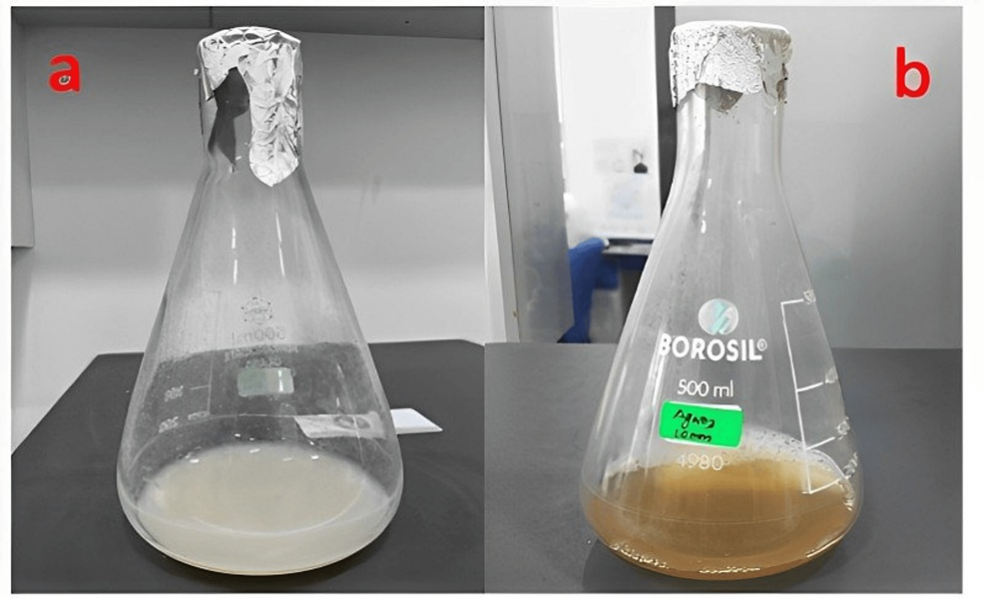
Synthesized silver nanoparticle (a) before (b) after

Silver nanoparticle synthesis: Silver nanoparticles were biosynthesized from silver nitrate by the actinobacterial isolate *Micromonospora* *sp*. MBIT-MSA4 (Figure 2). 

**Figure 3:**
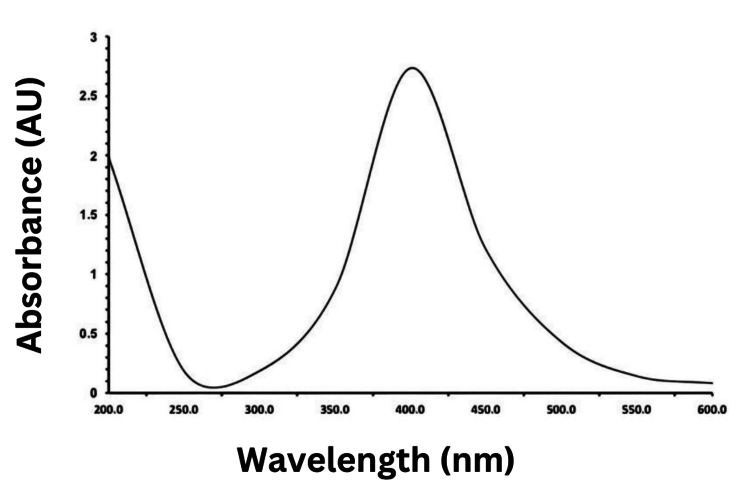
Ultraviolet spectrum of bio-synthesized silver nanoparticles by MBIT-MSA4

The biosynthesized silver nanoparticle was confirmed through the UV spectrum (Figure 3), which showed a peak at 450 nm. The presence of this peak in the UV-Vis spectrum indicates the absorption of light by silver nanoparticles (AgNPs) at that specific wavelength known as plasmon resonance. Plasmon resonance is a characteristic feature of silver nanoparticles. Typically, the peak falls within the range of 400 to 500 nm, with 450 nm being a common wavelength for this peak.

Antimicrobial activity

**Figure 4:**
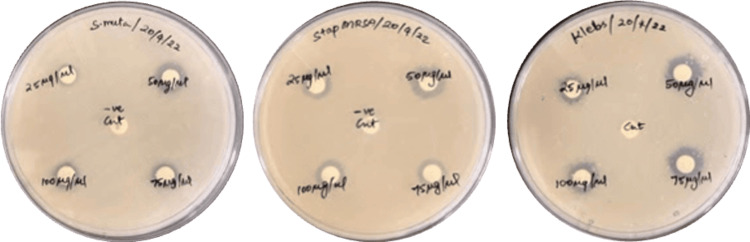
Antibacterial activity of silver nanoparticles against clinical pathogens. *Streptococcus mutans*, *Staphylococcus aureus, *and *Klebsiella pneumonia* multidrug resistant Strain (MRS).

The biosynthesized silver nanoparticle can inhibit clinical pathogens *Streptococcus mutans* (6 mm), *Klebsiella pneumonia* (10 mm), and S*taphylococcus aureus* (8 mm) (Figure 4). The total antioxidant activity has shown 73% inhibition of free radicals at a concentration of 50 𝜇g/mlof AgNP. Furthermore, the higher concentrations of 75-100 𝜇g/ml have not shown significant activity indicating the pro-oxidant level in the sample. 

Antioxidant activity

**Figure 5:**
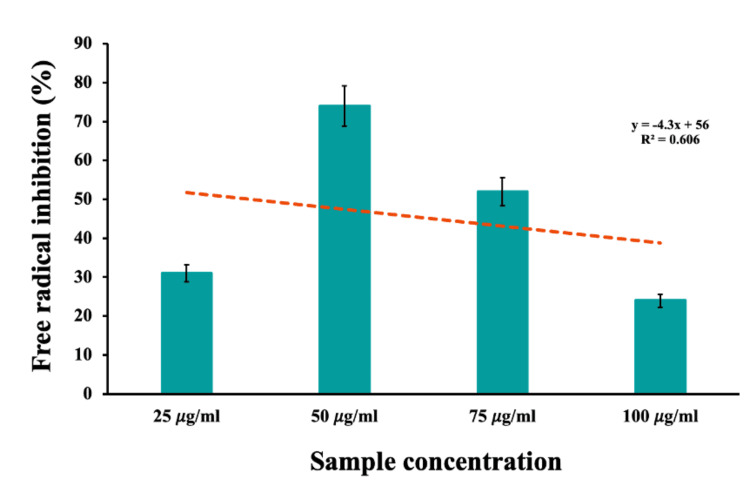
Antioxidant activity of biosynthesized silver nanoparticles

The concentration of the substance increases from 25 𝜇g/ml to 50 𝜇g/ml, resulting in a significant increase in antioxidant activity from 31% to 74% (Figure 5). This indicates that the AgNP is more efficient at neutralizing free radicals and protecting against oxidative damage as the concentration increases within this range. 

The dose-dependent response suggests that the substance has a positive impact on antioxidant activity. However, at a concentration of 75 𝜇g/ml, the antioxidant activity starts to decrease to 52% and there may be an optimal concentration range where the substance exhibits its maximum antioxidant potential. In this case, it appears that the peak antioxidant activity is reached at a concentration of 50 𝜇g/ml. Interestingly, at the highest concentration tested (100 𝜇g/ml), the antioxidant activity drops even further to 24%. This decline in antioxidant activity at the highest concentration could imply that there is a saturation point of the substance, beyond which it no longer provides antioxidant activity. Alternatively, it is possible that beyond this saturation point, the substance may have pro-oxidant effects, meaning that it could actually promote oxidative stress instead of reducing it. 

## Discussion

Detailed research has been conducted on the isolation, identification, and taxonomy of actinobacteria from various marine environments. Actinobacteria have attracted for their potential to produce novel compounds for the treatment of various diseases [21].

Rare genera of actinobacteria such as *Actinomyces*, *Nocardia,* and *Micromonospora, have been* isolated from the Sundarbans mangrove forest in West Bengal [22]. Similarly, a new species of *Streptomyces* *sp.* that produces an antimicrobial compound (iso-allyloxy phenyl) has been identified as *Streptomyces sundarbansensis* [23]. Similarly, the present study aims to isolate marine actinobacteria from sediment samples in Ennore, Chennai. Their resistance to silver nitrate was evaluated for further nanoparticle synthesis and potential isolates were identified using a polyphasic taxonomic method.

In the identification processes the composition of cell wall amino acids and sugars was crucial for determining the genus level. In this study, the presence of meso-DAP along with glycine indicates a cell wall type II suggesting that the isolates may belong to genera *Actinomyces, Actinoplanes, Ampulariella, Catellarosporia, Dactylosporangium, Glycomyces, Micromonospora, *and *Pilimelia*. Furthermore, the presence of arabinose and xylose in the cell wall sugar pattern D suggests the genera *Actinoplanes* and *Micromonospora*. Moreover, the formation of single spores with light orange-colored aerial mycelium indicates that the isolates belong to the genus *Micromonospora sp*. Silver nanoparticles derived from microbes have shown promise as a source of biosynthetic metabolites with significant antibacterial activity against various bacteria species. The secondary metabolites derived from *Micromonospora* *sp*. contain diverse bioactive compounds that possess antimicrobial, antioxidant, anticancer, and other biomedical properties [24]. 

The plasmon resonance peak occurs due to the collective oscillation of free electrons in the metal nanoparticles in response to incident light. This resonance phenomenon enhances the absorption of light at a specific wavelength, resulting in a peak in the UV-Vis spectrum [25]. The exact position of the plasmon resonance peak can vary depending on factors such as the size, shape, and surface properties of the nanoparticles. In the case of silver nanoparticles, the presence and position of the plasmon resonance peak are often used to characterize and confirm the formation of silver nanoparticles in various applications, including nanotechnology, materials science, and biology. It provides valuable information about the optical properties and size distribution of the nanoparticles [25]. 

Nanoparticles were biosynthesized from actinobacteria *Nocardiopsis sp*. marine biological research lab culture (MBRC-1), which possessed antimicrobial and cytotoxic properties. The biosynthesized silver nanoparticles exhibited a 29 mm inhibitory zone against *Streptococcus mutans *and a 15 mm inhibitory zone against *Staphylococcus aureus,* indicating a 92% inhibition rate [26]. Furthermore, they demonstrated potential antioxidant activity of 74% at a concentration of 50 𝜇g/ml reversing the processes. Similarly, Zhornik et al., [27] reported that silver nanoparticles induced lipid peroxidation in human lymphocytes, resulting in reduced cell viability. This suggests that the oxidation of silver nanoparticles through electron transfer may produce toxic substances.

The oxidation of silver ions leads to the formation of Ag_2_O or AgO, which generates oxygen (O_2_) and ozone (O_3_) as well as hydrogen peroxide (H_2_O_2_) radicals. These oxidized silver nanoparticles have an impact on antimicrobial and antioxidant activity. 

Limitation

The primary limitation of the study is the lack of standardized extraction techniques for silver nanoparticles from various sources. These techniques can impact the size, shape, stability, and bioactivity of the nanoparticles. Currently, there is no standardized specific protocol for extracting silver nanoparticles from microbes, plants, and other sources. It is crucial to address this limitation. Additionally, most of the studies conducted so far have been in vitro at the laboratory level. This lack of in vivo studies limits our understanding of the complexity of biosynthesized silver nanoparticles in living systems. Therefore, future research should focus on conducting in vivo experiments to gain a better understanding of the behavior of biosynthesized silver nanoparticles in living organisms.

## Conclusions

In conclusion, the biosynthesis of silver nanoparticles (AgNPs) from actinobacteria has yielded promising results in terms of their antimicrobial activity against *S. mutans* and methicillin-resistant *Staphylococcus aureus* (MRSA). Additionally, at specific concentrations, AgNPs have been found to exhibit antioxidant activity. However, higher concentrations have shown an oxidation effect, suggesting that AgNPs may undergo oxidation processes through electron transfer, potentially leading to the formation of toxic substances. This outcome depends on the physicochemical properties of AgNPs. The findings of the present study show that understanding the interactions between AgNPs and biological cells is essential for their safe use and further development. Proper assessment of the cytotoxic effects of AgNPs is important to ensure their potential benefits and avoid risks in medical and environmental applications. Further research should focus on the interaction of silver nanoparticles, and potential modifications to enhance their safety and efficacy. 
